# Prevalence of proprioception deficits in dogs visiting veterinary clinics for non-neurologic reasons: a cross-sectional study

**DOI:** 10.1093/jvimsj/aalag236

**Published:** 2026-10-09

**Authors:** Audrey Tam, Patrick Carney, Yael Merbl

**Affiliations:** Department of Clinical Sciences, College of Veterinary Medicine, Cornell University, Ithaca, NY 14853, United States; Department of Clinical Sciences, College of Veterinary Medicine, Cornell University, Ithaca, NY 14853, United States; Department of Clinical Sciences, College of Veterinary Medicine, Cornell University, Ithaca, NY 14853, United States

**Keywords:** degenerative, IVDD, general proprioception, geriatric dogs

## Abstract

**Background:**

General proprioception deficits (GPD) detected during neurological exams aid in neurologic assessment and steer practitioners to consider neurological differential diagnoses. However, prevalence of GPD in without clinical signs of neurologic disease is limited.

**Hypothesis/Objectives:**

That elderly dogs have GPD that might be undetected in dogs not presenting for neurologic concerns and to determine GPD prevalence in dogs presented to a veterinary teaching hospital.

**Animals:**

The study included 277 client-owned dogs presenting to the Cornell University without neurologic complaints.

**Methods:**

Cross-sectional study with prospective data collection involved neurological assessment of dogs. Two hundred seventy-seven dogs met the inclusion criteria. For statistical analysis, ages were categorized into fractional lifespan (FL) groups: Group A (adult, ≤ 0.5), Group B (middle-aged, 0.5-0.75), and Group C (senior, >0.75). Dogs were scored based on hopping and paw replacement performance as normal, mildly, or severely affected.

**Results:**

Seventy-two (26.0%; 95% CI, 20.9-31.7) exhibited GPD while 205 (74.0%) did not. Age distribution revealed 102 dogs (36.8%) in FL category 1 (≤0.5), 70 dogs (25.3%) in category 2 (>0.5 to ≤ 0.75), and 105 dogs (37.9%) in category 3 (>0.75). Both age (odds ratio [OR] 1.22, 95% CI, 1.13-1.33) and FL (OR 1.31, 95% CI, 1.18-1.46) were significantly positively associated with GPD (*P* < .0001).

**Conclusions and clinical importance:**

The prevalence of incidental GPD in dogs with no known neurological disease increases with age and is not influenced by sex or chondrodystrophic status. Awareness of this finding might aid general practitioners’ decision-making, especially in aging dogs without overt acute neurologic disease.

## Introduction

Proprioception, the body’s subconscious awareness of its position, orientation, and movement, is essential for normal gait and posture. It relies on central integration of afferent signals from muscle spindles, joint receptors, and tactile receptors, together with efferent output from upper motor neuron centers.^1^^,^^2^

Evaluation of proprioception is an important component of a complete neurologic examination and involves assessment of posture, gait, and postural reactions such as paw replacement and hopping.^2^ General proprioception deficits (GPD) can result from lesions anywhere along the proprioceptive pathway, from peripheral receptors to the brain. Postural reactions, evaluated alongside other neurologic findings, provide valuable information for localizing a lesion, generating differential diagnoses, and guiding diagnostic and treatment recommendations.

GPD are particularly relevant to (1) distinguishing multifocal from multiple unrelated disease processes, and (2) differentiating central from peripheral vestibular disease, since a peripheral lesion should not cause postural reaction deficits. Although the neurologic examination reliably localizes vestibular disease, GPD occur in up to 73% of vestibular dogs with lesions outside the brain.^3^ This raises the question of whether incidental GPD are under-recognized. In one study of dogs > 7 years old, 9 out of 21 had proprioceptive deficits unnoticed by owners who considered them “neurologically normal.”^4^

Understanding the baseline prevalence of incidental GPD has direct clinical relevance: overinterpreting; as an incidental postural reaction deficit as active neurologic disease could prompt unnecessary diagnostics or client concern, whereas dismissing it as an inevitable feature of aging could mean overlooking early progressive disease. Establishing the expected background prevalence of GPD across age groups would allow practitioners to calibrate their interpretation of such findings rather than relying on subjective impressions of “normal” aging.

Although it is a common perception that dogs might also show proprioceptive deficits as they age, no study to date has specifically investigated the prevalence of proprioceptive deficits in dogs with no reported neurologic signs. In this study, we aimed to explore the relationship between incidental proprioceptive deficits, age, sex, and breed. We hypothesized that prevalence of proprioceptive deficits would increase with age and would be more common in chondrodystrophic breeds.

## Materials and methods

This cross-sectional study with prospective data collection captured neurological examination findings during physical examinations of dogs presenting to (masked for review) between May and July 2025. This 3-month enrollment window was selected as a period of convenience based on examiner and student availability, rather than a predetermined study duration. The protocol was deemed IACUC-exempt; verbal informed consent was obtained from all owners. A study flow diagram is provided in Figure 1.

**Figure 1:**
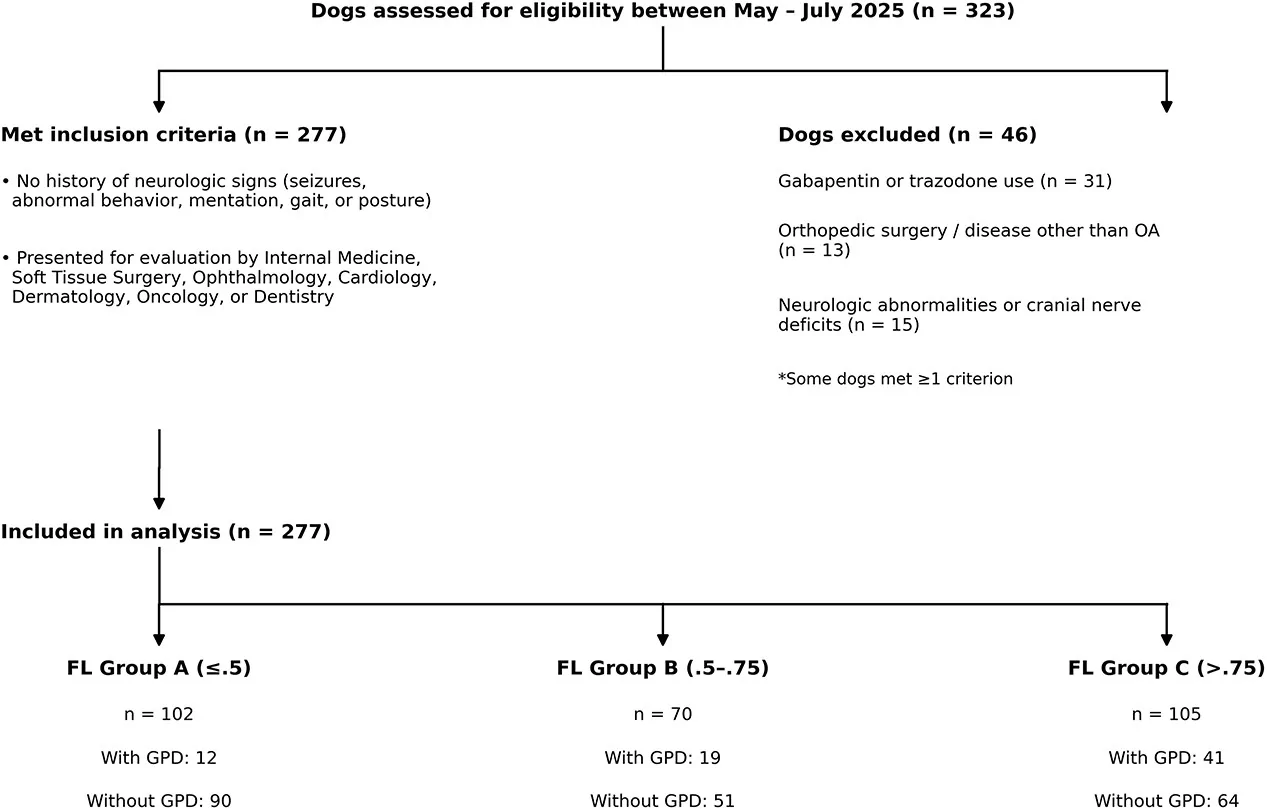
Study flow diagram. GPD = general proprioceptive deficit. OA = osteoarthritis. Exclusion counts sum to > 46 because some dogs met more than one criterion. FL = fractional lifespan; FL groups are defined as in Table 2 (Group A, FL ≤ 0.5; Group B, FL 0.5-0.75; Group C, FL > 0.75). Dates of enrollment (May–July 2025).

Client-owned dogs were eligible if owners reported no history of neurologic signs (seizures, abnormal behavior, mentation, gait, or posture) and they presented for evaluation by Internal Medicine, Soft Tissue Surgery, Ophthalmology, Cardiology, Dermatology, Oncology, or Dentistry. Two veterinary students and one rotating intern, trained by a neurology resident until consensus was reached on abnormal findings, performed neurologic examinations (cranial nerves, spinal reflexes, limb tone, posture, and postural reactions) while blinded to each dog’s age, using a standardized evaluation sheet (Supplementary Material S1). Reflexes and tone were graded 0 (normal) to 2 (increased); postural reactions (hopping, paw replacement) were graded 0 (normal) to 3 (absent), with grades 1-3 dichotomized as abnormal for the primary analysis. Height (floor to withers), signalment, weight, medications, and comorbidities were also recorded.

Fractional lifespan (FL) was calculated as each dog’s age divided by its estimated lifespan,^5^ derived from weight, age, and height using the formula of Greer et al.^6^ Dogs were grouped as FL ≤ 0.5 (Group A, adult), 0.5-0.75 (Group B, middle-aged), or >0.75 (Group C, senior). Target enrollment (*n* = 250) was based on 95% CI width around prevalence estimates; formal power analysis was not performed. Dogs were excluded for prior orthopedic surgery or orthopedic disease other than osteoarthritis, recent or chronic gabapentin or trazodone use,^7^^,^^8^ or cranial nerve abnormalities. Breeds with a reported CDDY mutation frequency ≥ 50% were classified CDDY = 1; all others, including mixed breeds, were classified CDDY = 0. Full methodological detail is provided in Appendix.

### Statistical analysis

Data were analyzed using SAS 9.4 (Cary, NC). Statistical analyses included chi-square, Fisher’s exact, Fisher–Freeman–Halton, Wilcoxon rank-sum, and bivariate and multivariate logistic regression (including Firth’s penalized likelihood where indicated) to assess associations between GPD and age, FL, sex, neuter status, and CDDY status, with Benjamini-Hochberg correction for multiple comparisons. Significance was set at *P* < .05. Full statistical methodology is provided in Appendix.

## Results

### Animals

A total of 323 dogs were examined during the study period. Of these, 277 met the inclusion criteria. Forty-six dogs were excluded due to administration of gabapentin or trazodone (*n* = 31), history of orthopedic surgery or orthopedic disease other than osteoarthritis (*n* = 13), history of neurologic abnormalities or cranial nerve deficits in the absence of ocular disease (*n* = 15), or a combination of multiple of these characteristics. Full data demographics are summarized in Table 1.

**Table 1:** Demographic characteristics of study cohort (*n* = 277).

Characteristic	n	%
**Sex and reproductive status**		
**Spayed Female**	119	43.0
**Neutered Male**	114	41.2
**Intact Male**	21	7.6
**Intact Female**	23	8.3
**Total Intact**	44	15.9
**Total Neutered/Spayed**	233	84.1
**Age at examination (years)**		
**Median (IQR)**	7.0 (4.0-10.0)	
**Range**	0-15	
**0-3 years**	51	18.4
**4-6 years**	66	23.8
**7-9 years**	68	24.5
**10-12 years**	67	24.2
**≥13 years**	25	9.0
**Body weight (lbs)**		
**Median (IQR)**	38.8 (17.5-64.5)	
**Range**	3.3-140.0	
**Breed**		
**Beagle/Beagle Hound**	5	1.8
**Boston Terrier**	5	1.8
**Boxer**	4	1.4
**Chihuahua**	5	1.8
**Cavalier King Charles Spaniel**	10	3.6
**English Bulldog**	11	4.0
**French Bulldog**	10	3.6
**German Shepherd Dog**	4	1.4
**Golden Retriever**	15	5.4
**Husky**	7	2.5
**Labrador Retriever**	27	9.7
**Toy/Miniature Poodle**	8	2.9
**Pembroke Welsh Corgi**	4	1.4
**Shih Tzu**	11	4.0
**Yorkshire Terrier**	5	1.8
**Mixed**	74	26.7
**Other (≤3 each)**	72	26.0
**Fractional Lifespan**		
**Median (IQR)**	0.592 (0.362-0.827)	
**Range**	0.000- 1.128	

Abbreviation: IQR = interquartile range. “Other” includes breeds with ≤ 3 individual cases. Age range 0-15 years; the minimum of 0 reflects a dog less than 1 month old at examination (FL reported as 0.000), who met all inclusion criteria and presented for a non-neurological appointment. Breeds assigned CDDY = 1 (frequency ≥ 50% per UC Davis VGL database) included: Beagle, Boston Terrier, English Bulldog, French Bulldog, Cavalier King Charles Spaniel, Pembroke Welsh Corgi, Shih Tzu, Dachshund, and other breeds with published CDDY frequency ≥ 50%.

### General proprioceptive deficits

#### Age distribution among sex groups

Age significantly varied between sex groups (*P* < .0001). Intact dogs were significantly younger than neutered dogs, but that there were no statistically significant age differences between intact males and females, or between neutered males and females.

#### General proprioceptive deficits

Of the 277 dogs included in the study, 72 (26.0%; 95% CI, 20.9-31.7) had delayed postural reactions in at least one limb. The pelvic limbs were the most affected, with 65 out of 72 dogs (90%) having only pelvic limb deficits, 6 out of 72 dogs (8%) being affected in thoracic and pelvic limbs, and 1 out of 72 dog (1%) with only one thoracic limb affected. Fifteen dogs (21%) had deficits in one limb only; 14 out of 15 (93%) had a deficit in a pelvic limb. The remaining 57 dogs with deficits were affected in either both pelvic limbs or all 4 limbs.

Thirty-one out of 72 (43%) dogs with postural reaction deficits had delayed “hopping” only whereas 3 out of 72 (4%) had delayed paw replacement only. The remainder (38/72, 52%) had both delayed hopping and paw replacement. Spinal reflexes (withdrawal, patellar, cutaneous trunci) were normal in all dogs with proprioceptive deficits. Only one dog, which had deficits in its pelvic limbs, had higher pelvic limb tone.

##### General proprioception deficits distribution based on age

In bivariate logistic regression, age was significantly positively associated with the presence of proprioceptive deficits (*P* < .0001). For each additional year of age, the odds of proprioceptive deficits increased by 22% (ie, OR 1.22, 95% CI, 1.13-1.33).

##### General proprioception deficits based on fractional lifespan category

In bivariate logistic regression, FL was significantly positively associated with the presence of proprioceptive deficits (*P* < .0001). For each additional 0.1 FL, the odds of proprioceptive deficits increased by 31% (ie, OR 1.31, 95% CI, 1.18-1.46). Dogs with proprioceptive deficits were significantly older than those without (*P* < .0001), with dogs having proprioceptive deficits having a median FL of 0.82 (IQR 0.35, range 0.09-1.15) and dogs without proprioceptive deficits having a median FL of 0.54 (IQR 0.48, range 0.01-1.13).

The FL category and GPD were significantly associated (*P* < .0001) (Table 2, Figure 2). Post-hoc Fisher’s exact test with Benjamini-Hochberg correction found that the prevalence of proprioceptive deficits differed significantly between FL groups A and B (*P* = .0048) and between A and C (*P* < .0003), but not between B and C (*P* = .3317).

**Table 2:** GPD prevalence by fractional lifespan group.

FL Group	Total (*n*)	With GPD (*n*)	95% CI	% With GPD	% Without GPD
**A (≤0.5)**	102	12	6.9-19.4	11.8	88.2
**B (0.5-0.75)**	70	19	18.1-38.5	27.1	72.9
**C (>0.75)**	105	41	30.3-48.6	39.0	61.0

Abbreviations: FL = fractional lifespan; GPD = general proprioception deficit. 95% CIs are Wilson score intervals. Post-hoc pairwise *P*-values (Benjamini-Hochberg corrected): A vs. B *P* = .0048*; A vs. C *P* < .0003*; B vs. C *P* = .3317.

**Figure 2:**
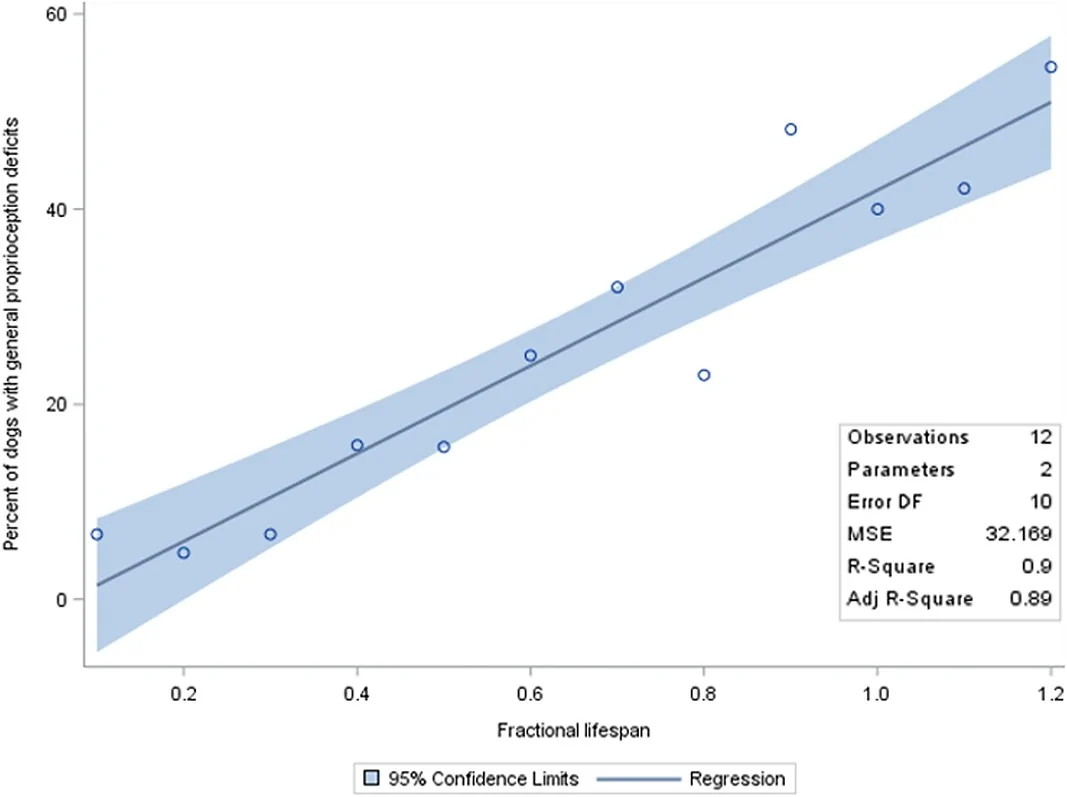
Association between FL and GPD. The relationship between FL and the percentage of dogs with proprioceptive deficits was significant (F 90.4, 1 d.f., *P* < .0001), r^2^ = 0.90. Normality of residuals was assessed via visual inspection of normal QQ plot, and homoscedasticity was assessed by visual inspection of residual versus predicted plot.

##### Distribution of proprioceptive deficits by sex

In bivariate analysis, the distribution of proprioceptive deficits differed by sex (*P* = .0049). In false discovery rate-corrected post-hoc analysis, female intact dogs had significantly lower GPD prevalence than female spayed (FS) (*P* = .0200), male intact (MI) (*P* = .0308), and male neutered (*P* = .0030) dogs. However, this effect is likely mostly mediated by age, as shown by logistic regression using Firth’s penalized likelihood including both FL and sex to predict proprioceptive deficits, where FL was a significant predictor of proprioceptive deficits (OR 12.09 [3.98-36.70], *P* < .0001) but sex was not (*P* = .2303).

Sex alone and neuter status were individually investigated to better understand their relationship to proprioceptive deficits. In chi square analysis, sex alone was not significantly associated with proprioceptive deficits (relative risk [female vs. male] 0.679 [0.453, 1.018], *P* = .0583). In chi square analysis, neuter status was significantly associated with proprioceptive deficits (relative risk [intact vs. neutered] 0.481 [0.223, 1.04], *P* = .0416). Both are likely confounded by age; in multivariate logistic regression, FL was associated with proprioceptive deficits (OR 12.80 [4.24, 38.67], *P* < .0001), while neither sex alone (*P* = .1122) nor neuter status (*P* = .4772) was significantly associated.

Looking at proprioceptive deficits by sex (with neuter status) and by FL category using multivariate logistic regression, sex was not a significant predictor (*P* = .2377) while FL category was (*P* < .0008), again demonstrating that the bivariate association between sex and proprioceptive deficits noted previously is likely confounded by age.

### Breed: chondrodystrophic versus non-chondrodystrophic

CDDY breed status was not significantly associated with GPD (OR 1.02 [0.47, 2.22], *P* = .9609).

### Age distribution among sex groups

Age significantly varied between sex groups (*P* < .0001). Intact dogs were significantly younger than neutered dogs, but that there were no statistically significant age differences between intact males and females, or between neutered males and females.

## Discussion

In this study we sought to explore the relationship between age and GPD in a cohort of dogs not suspected of having neurologic disease. We confirmed that a substantial proportion of dogs might have GPD that are unnoticed by owners and that GPD prevalence increases with both age and FL. FL was assessed as a means of accounting for breed size differences in expected longevity (ie, a 10-year-old Great Dane might be considered “older” than a 10-year-old Chihuahua).

A quarter of our study cohort had postural reaction deficits in at least one limb. This is consistent with findings of 2 previous studies recruiting dogs with no reported neurologic abnormalities that showed 7 out of 32 dogs between 1-6 years of age had incidental GPD^8^ and 9 out of 21 dogs greater than 7 years of age had GPD.^4^ Although these studies were not specifically investigating the prevalence of proprioceptive deficits, their results support the present finding that GPD are likely under-recognized as incidental examination findings.

In our study, 43.1% of affected dogs had solely delayed hopping and 4.2% had solely delayed paw replacement. This finding might suggest that hopping could be a more sensitive test for subclinical proprioceptive deficits in this cohort. Hopping assessment is more dependent on dynamic proprioceptive changes by shifting the animal’s center of gravity over a single limb, recruiting the muscle spindle, Golgi tendon organ, and joint receptor inputs simultaneously. Paw replacement assessment, by contrast, relies primarily on tactile receptor activation. The higher rate of isolated hopping deficits could portray early dysfunction of the more complex proprioceptive pathway, while tactile-mediated paw replacement responses remain intact. We suggest that paw replacement responses alone might underestimate the true prevalence of proprioceptive impairment, and that hopping assessment of all 4 limbs should be included in routine neurologic screening of older dogs.

The presence of GPD was significantly associated with age and FL, with the odds of GPD increasing with both age and FL. Numerous studies in humans report lower proprioception, joint position sense, and postural control in older adults.^9^^,^^10^ This might be due to aging-correlated changes in the number^11^ and diameter of muscle spindle fibers,^12^ differences in spinal synaptic integration,^13^ structural changes in sensorimotor brain regions,^14^^,^^15^ and alterations in neurotransmitter concentrations and brain activation.^16^^,^^17^ Similarly, in mice and rabbits have reductions in the number of joint and tendon mechanoreceptors^18^ and peripheral nerve fibers^19^ in older animals that could potentially affect proprioception. Postural control is impaired in older dogs.^20^ The study reported that postural sway increased with aging and was associated with a higher FL in senior (FL > 0.75) but not younger dogs. Among senior dogs, 9 out of 24 dogs had reduced postural reactions and those with abnormal proprioception had a higher FL than those without. Although the presence of GPD alone was not correlated with postural sway, the interaction of joint pain and GPD did influence postural sway, suggesting that proprioception might play an important role in postural control in dogs as it does in humans.

The findings of our study suggest that a FL of 0.5 might represent a clinically relevant threshold above which the likelihood of incidental GPD increases substantially. This period corresponds to the transition from the adult to middle-aged life stage. During this period, routine neurologic screening, including assessment of postural reactions, might be warranted as part of wellness examinations, even in the absence of owner-reported neurologic concerns. Dogs with a FL exceeding 0.75 had a GPD prevalence above 40%, suggesting that neurologic screening in this cohort might be beneficial.

None of the dogs with GPD in the present study had abnormal spinal reflexes and only one had changes in limb tone (increased). This could support the hypothesis that age-related changes in receptors, muscle fibers, synaptic integration, or brain activation might contribute to GPD in addition to lesions affecting spinal cord pathways. Alternatively, it is also possible that some dogs had early spinal cord lesion that was important enough to cause subtle GPD but was not severe enough to result in changes to reflexes or tone.

Pelvic limbs were more likely to be affected than thoracic limbs, with 90.3% of dogs with GPD having only pelvic limb deficits versus 1.4% with only thoracic limb deficits. The high prevalence of pelvic limb deficits might be consistent with age-related spinal cord lesions such as degenerative conditions affecting the thoracolumbar and lumbosacral regions including degenerative myelopathy, lumbosacral spondylosis deformans, and intervertebral disc disease (IVDD). Chronic IVDD has a higher likelihood of affecting the more superficial pelvic limb proprioceptive spinal cord tracts than the more medially located thoracic limb tracts.^21^ The other mentioned diseases mainly affect the caudal spinal cord and its long ascending proprioceptive tracts, which are responsible for pelvic limb function. The pelvic limbs also have longer peripheral nerve pathways, potentially making them more susceptible to subclinical peripheral neuropathy. Human studies on aging and proprioception are biased toward investigations of the lower limb and have not explored potential differences in the upper and lower limbs.^10^ However, older adults are less sensitive to motion of metatarsophalangeal joints than metacarpophalangeal joints whereas there is no difference in joint motion perception in younger adults.^22^

Breed chondrodystrophy genetic frequency was not associated with GPD in our study. The CDDY mutation is associated with premature degeneration and calcification of intervertebral discs and a higher risk of intervertebral disc herniation.^23^^,^^24^ None of the dogs studied showed signs suggestive of a disc herniation. However, the CDDY cohort might have been underrepresented as dogs with a history of neurological episodes were excluded from enrollment. In addition, this study was designed to recognize slowly progressive cases that might otherwise go unnoticed, whereas dogs with CDDY-associated disease might be more likely to present with acute and dramatic clinical signs. The findings suggest that chondrodystrophic breeds that do not develop overt IVDD are not more likely than other breeds to develop subtle age-related GPD. Furthermore, CDDY status was inferred based on breed rather than confirmed genetically. Because breed-associated risk does not directly correspond to individual genotype, it is unlikely that all dogs of CDDY-associated breeds carried the mutation, which might have attenuated any association between chondrodystrophy and GPD. Mixed-breed dogs, which comprised a substantial portion of the study cohort (27.1%), were classified as CDDY-negative. The absence of confirmed genetic status in these dogs might have further influenced the analysis.

Our study had several other limitations. Data collection occurred at a veterinary teaching hospital and might not fully represent the general dog cohort. Dogs presenting to academic facilities might have higher rates of comorbidity, better owner’s engagement and different breeds ratios compared to dogs attending general practice. These factors could affect our observed GPD prevalence estimates. Additionally, the dogs in our study were presented to the veterinary hospital for appointments with specialty services. Although reported to have no orthopedic or neurologic abnormalities by their owners, they could have had other diagnosed and undiagnosed comorbidities that could have affected the neuroexamination findings. Many dogs had ocular disease that prevented accurate evaluation of a menace response and pupillary light reflexes. These dogs were assumed to have abnormal exams secondary to ocular changes but could have had obscure neurologic dysfunction. Furthermore, data collection was performed by one rotating intern and 2 veterinary students trained by the same neurology resident. Although the observers reached a consensus on abnormal findings during their standardized training period, formal inter-examiner reliability metrics were not calculated. Since postural reaction grading involves subjective assessment, some degree of inter-examiner variability cannot be excluded. It is reported that examiner experience can influence the detection and grading of postural reaction deficits, and that neurological specialists apply more nuanced clinical judgment than less experienced examiners.^3^^,^^25^ However, the use of examiners whose training and proficiency level approximates that of general practitioners who, while competent in performing a neurological examination, might not routinely screen for subtle proprioceptive deficits or might lack the confidence to attribute them to age-related changes rather than active, progressive neurological disease. The primary clinical goal of this work was not to characterize GPD at the level of specialist precision, but rather to demonstrate that such deficits are detectable by non-specialist examiners and that their prevalence increases with age. This distinction is clinically important: if general practitioners are to be encouraged to incorporate neurological screening into routine wellness examinations for older dogs, evidence generated using examiner profiles similar to their own is directly applicable. In addition, we used FL to account for differences in aging between different sizes of dogs, but the equation used was based on data from purebred dogs.^6^ In the present study, this equation was applied to both purebred and mixed breed dogs. Finally, the cross-sectional nature of this study precludes conclusions about whether GPD in individual dogs is progressive, stable, or intermittent over time. Longitudinal follow-up of dogs identified to have incidental GPD would be valuable to determine the natural history of these findings and their association with eventual neurologic disease.

In conclusion, our study explores the prevalence of “incidental” GPD and to demonstrate its association with aging. The prevalence of GPD increases with age and is unrelated to sex or chondrodystrophic status. Our findings should not be attributed to an active neurologic condition. Conversely, awareness of the expected background prevalence of GPD in aging dogs might help practitioners avoid normalizing findings in dogs that have signs of early progressive neurologic disease. This finding might aid practitioners in interpreting neurologic examination findings and can guide diagnostics and treatment recommendations, especially in an older dogs.

## Off-label antimicrobial declaration

Authors declare no off-label use of antimicrobials.

## Institutional animal care and use committee or other approval declaration

Authors declare no institutional animal care and use committee or other approval was needed.

## Appendix

### Detailed materials and methods

This appendix provides the complete methodological detail supporting the condensed Materials and Methods and Statistical Analysis sections of the main manuscript. Reference numbers correspond to the numbered reference list in the main manuscript.

### Study design, setting, and ethical oversight

This study used a cross-sectional design with prospective data collection, capturing neurological examination findings as part of the physical examination of dogs presenting to Cornell University Hospital for Animals for appointments between May and July 2025. The experimental protocol was reviewed by the IACUC committee and deemed exempt, as the data entailed only information collected during a physical examination, which is a common and expected practice. Informed consent was verbally obtained from all owners. A flow diagram illustrating the inclusion and exclusion process is provided as Figure 1.

Client-owned dogs were recruited for the study if (1) no history of neurologic signs (seizures, abnormal behavior, mentation, gait, or posture) were reported by owners, and (2) they were presented for evaluation by any of the following services: Internal Medicine, Soft Tissue Surgery, Ophthalmology, Cardiology, Dermatology, Oncology, or Dentistry.

### Examiner training and neurologic examination procedures

Two veterinary students and one rotating intern were trained by the same neurology resident on how to perform the neurologic examination, and data collection began once a consensus was reached on abnormal findings. More specifically, the neurology resident training the examiners looked at several dogs with each trainee to refine the exam and be satisfied that all examiners perceived and interpreted the deficits in the same manner. The examiners were blinded to the age of each dog and recorded examination results using a standard clinical evaluation sheet.

The neurologic examinations performed included evaluation of cranial nerves, spinal reflexes, limb tone, posture, and postural reactions. Reflexes and muscle tone were graded as follows: 0, normal; 1, decreased; 2, increased. Postural reactions (hopping and paw replacement) were graded on a scale from 0 to 3 as follows: 0, normal; 1, mildly delayed (1 s); 2, moderately to severely delayed (2+ seconds); 3, absent. Hopping was performed by holding the dog with its weight supported by one limb, moving it laterally, and observing how quickly it replaced the limb. Paw replacement was tested by supporting the dog, turning one paw over to place its dorsal surface in contact with the ground, and evaluating time to replacement of the paw.

Gait was evaluated by the examining clinician as part of the neurological examination; owner-reported gait history was used as an inclusion criterion to exclude dogs with known neurological complaints. As any dog with clinically abnormal gait would have met exclusion criteria, gait was not included as a primary outcome variable. The height of each dog was measured from the floor to the dorsal border of the scapula (withers). Signalment, weight, current medications, and comorbidities were recorded for each subject after the examination.

### Fractional lifespan calculation and sample size justification

Due to the effects of body size on lifespan in dogs,^5^ the lifespan of each dog was estimated using the formula described by Greer et al.,^6^ which takes into account weight, age, and height. Each dog’s age at the time of the study was divided by its estimated lifespan to generate “fractional lifespan” (FL), which ranges from 0 (birth) to > 1 (exceeding its predicted lifespan), for statistical comparison. Dogs with a FL ≤ 0.5 were classified as group A (adult), those with FL between 0.5 and 0.75 were included in group B (middle-aged), and those with FL > 0.75 were classified as group C (senior).

The enrollment period was chosen based on a target of *n* = 250, which was determined from graphs of 95% confidence limits around prevalence estimates of 1%-40% that were generated for *n* = 100, 150, 200, 250, and 300. Negligible narrowing of the confidence intervals was seen going from 250 to 300. As the primary goal of the study was to determine a proportion, formal power analysis was not performed; however, this is acknowledged as a limitation.

### Exclusion criteria and breed classification

Dogs were excluded prior to data analysis if they had a history of orthopedic surgery or had been diagnosed with orthopedic conditions aside from osteoarthritis, and if they had received gabapentin or trazodone either chronically or on the day of their appointment, as both medications have been reported to cause ataxia or postural reaction deficits in dogs.^7^^,^^8^ Dogs that had abnormalities on their cranial nerve exam were also excluded, as an intracranial cause of GPD could not be ruled out.

Dogs were categorized based on the published frequency of the chondrodystrophy mutation (CDDY) reported by the UC Davis Veterinary Genetics Laboratory (https://vgl.ucdavis.edu/test/cddy-cdpa). Dogs belonging to breeds with a CDDY frequency ≥ 50% were assigned CDDY = 1, while all others (including mixed breeds) were assigned CDDY = 0.

### Statistical analysis

Data were analyzed using SAS 9.4 (Cary, NC). Categorical variables were summarized by counts and percentages. The Shapiro–Wilk test was used to assess continuous variables for normality; approximately normally distributed variables were reported as mean and standard deviation, while non-normal variables were described via median and IQR.

One-way ANOVA with Tukey’s HSD post-hoc test was used to assess the relationship between sex and age, with homogeneity of variances checked via Levene’s test and normality of residuals via visual inspection of the residual QQ plot. Chi square analysis was used for the association between FL category and GPD, with post-hoc Fisher’s exact test with Benjamini-Hochberg correction. For the purposes of the primary analysis, postural reaction grades were dichotomized into normal (grade 0) versus abnormal (grades 1-3) to maintain statistical power and clinical simplicity; the detailed distribution of grading scores is provided in the Supplementary Material (S1).

More granular FL categories (0.1-unit bins, 12 total bins) were generated to assess how the percentage of subjects with GPD changed with lifespan, with normality of residuals assessed via visual inspection of the normal QQ plot and homoscedasticity assessed via residual versus predicted plot. The association between GPD and FL as a continuous variable was explored using bivariate logistic regression and the Wilcoxon rank sum test. Bivariate logistic regression was used to examine the association between GPD and age, and the association between chondrodystrophy risk and GPD.

The relationship between sex and GPD was assessed by the Fisher–Freeman–Halton test (the extension of Fisher’s exact test for r × c tables), which was appropriate given the multi-level sex variable; Benjamini-Hochberg correction was applied to post-hoc pairwise comparisons. The Benjamini-Hochberg false discovery rate procedure was also applied to the 3 pairwise post-hoc comparisons between FL groups (A vs. B, A vs. C, B vs. C) as a transparent and accepted method for controlling multiplicity in exploratory analyses.

Body weight was not included as an independent predictor, as both weight and height contribute to the FL estimate; including body weight alongside FL would introduce co-linearity. Multivariate logistic regression was used to assess covariate-adjusted relationships of FL, sex alone, and neuter status with GPD. When the intact female subgroup exhibited near-separation in standard maximum likelihood logistic regression, Firth’s penalized likelihood method was used. The Box-Tidwell test was used to evaluate the assumption of linearity of independent variables and log odds. Statistical significance was established at *P* < .05.

## Abbreviations

CI: confidence interval
FI: female intact
FL: fractional lifespan
FS: female spayed
GPD: general proprioception deficits
MI: male intact
MN: male neutered
IQR: interquartile range
OR: odds ratio
SD: standard deviation

## References

[1] Boisgontier MP, Olivier I, Chenu O, Nougier V. Presbypropria: the effects of physiological ageing on proprioceptive control. AGE. 2012;34:1179–1194. 10.1007/s11357-011-9300-y PMC3448996 21850402

[2] Thomson C, Hahn C. Veterinary Neuroanatomy: A Clinical Approach. Saunders Elsevier; 2012.

[3] Boudreau CE, Dominguez CE, Levine JM, et al. Reliability of interpretation of neurologic examination findings for the localization of vestibular dysfunction in dogs. JAVMA. 2018;252:830–838. 10.2460/javma.252.7.830 29553894

[4] Golini L, Colangeli R, Tranquillo V, Mariscoli M. Association between neurologic and cognitive dysfunction signs in a sample of aging dogs. J Vet Behav. 2009;4:25–30. 10.1016/j.jveb.2008.09.033

[5] Urfer SR, Kaeberlein M, Promislow DEL, Creevy KE. Lifespan of companion dogs seen in three independent primary care veterinary clinics in the United States. Canine Med Genet. 2020;7:7. 10.1186/s40575-020-00086-8 PMC7386164 32835231

[6] Greer KA, Canterberry SC, Murphy KE. Statistical analysis regarding the effects of height and weight on life span of the domestic dog. Res Vet Sci. 2007;82:208–214. 10.1016/j.rvsc.2006.06.005 16919689

[7] Di Cesare F, Negro V, Ravasio G, Villa R, Draghi S, Cagnardi P. Gabapentin: clinical use and pharmacokinetics in dogs, cats, and horses. Animals. 2023;13:2045. 10.3390/ani13122045 PMC10295034 37370556

[8] Lueck LC, Cameron S, Zidan N. Effects of trazodone administration on the neurologic examination in healthy dogs. J Vet Intern Med. 2022;36:2142–2148. 10.1111/jvim.16536 PMC9708436 36086912

[9] Henry M, Baudry S. Age-related changes in leg proprioception: implications for postural control. J Neurophysiol. 2019;122:525–538. 10.1152/jn.00067.2019 PMC6734411 31166819

[10] Goble DJ, Coxon JP, Wenderoth N, Van Impe A, Swinnen SP. Proprioceptive sensibility in the elderly: degeneration, functional consequences and plastic-adaptive processes. Neurosci Biobehav Rev. 2008;33:271–278. 10.1016/j.neubiorev.2008.08.012 18793668

[11] Liu JX, Eriksson PO, Thornell LE, Pedrosa-Domellöf F. Fiber content and myosin heavy chain composition of muscle spindles in aged human biceps brachii. J Histochem Cytochem. 2005;53:445–454. 10.1369/jhc.4A6257.2005 15805419

[12] Kararizou E, Manta P, Kalfakis N, Vassilopoulos D. Morphometric study of the human muscle spindle. Anal Quant Cytol Histol. 2005;27:1–4. 15794446

[13] Terao S, Sobue G, Hashizume Y, Li M, Inagaki T, Mitsuma T. Age-related changes in human spinal ventral horn cells with special reference to the loss of small neurons in the intermediate zone: a quantitative analysis. Acta Neuropathol. 1996;92:109–114. 10.1007/s004010050497 8841655

[14] Boisgontier MP, Cheval B, Chalavi S, et al. Individual differences in brainstem and basal ganglia structure predict postural control and balance loss in young and older adults. Neurobiol Aging. 2017;50:47–59. 10.1016/j.neurobiolaging.2016.10.024 27875755

[15] Good CD, Johnsrude IS, Ashburner J, Henson RNA, Friston KJ, Frackowiak RSJ. A voxel-based morphometric study of ageing in 465 normal adult human brains. NeuroImage. 2001;14:21–36. 10.1006/nimg.2001.0786 11525331

[16] Goble DJ, Coxon JP, Van Impe A, et al. The neural basis of central proprioceptive processing in older versus younger adults: an important sensory role for right putamen. Hum Brain Mapp. 2012;33:895–908. 10.1002/hbm.21257 PMC6870471 21432946

[17] Gottfries CG . Neurochemical aspects on aging and diseases with cognitive impairment. J Neurosci Res. 1990;27:541–547. 10.1002/jnr.490270415 2079715

[18] Morisawa Y . Morphological study of mechanoreceptors on the coracoacromial ligament. J Orthop Sci. 1998;3:102–110. 10.1007/s007760050029 9654563

[19] Verdu E, Ceballos D, Vilches JJ, Navarro X. Influence of aging on peripheral nerve function and regeneration. J Peripher Nerv Syst. 2000;5:191–208. 10.1046/j.1529-8027.2000.00026.x 11151980

[20] Mondino A, Wagner G, Russell K, et al. Static posturography as a novel measure of the effects of aging on postural control in dogs. PloS One. 2022;17:e0268390. 10.1371/journal.pone.0268390 PMC9269968 35802714

[21] de Lahunta A, Glass E, Kent M. Small Animal Spinal Cord Disease. In: de Lahunta A, Glass E, Kent M, eds. De Lahunta’s Veterinary Neuroanatomy and Clinical Neurology. 5th ed. Elsevier Inc; 2020:267-311.

[22] Kokmen E, Bossemeyer RW, Williams WJ. Quantitative evaluation of joint motion sensation in an aging population. J Gerontol. 1978;33:62–67. 10.1093/geronj/33.1.62 618968

[23] Batcher K, Dickinson P, Giuffrida M, et al. Phenotypic effects of FGF4 retrogenes on intervertebral disc disease in dogs. Genes. 2019;10:435. 10.3390/genes10060435 PMC6627552 31181696

[24] Brown EA, Dickinson PJ, Mansour T, et al. FGF4 retrogene on CFA12 is responsible for chondrodystrophy and intervertebral disc disease in dogs. Proc Natl Acad Sci U S A. 2017;114:11476–11481. 10.1073/pnas.1709082114 PMC5664524 29073074

[25] Gonçalves R, Maddox TW, Phillipps S, et al. Development of a reliable clinical assessment tool for meningoencephalitis in dogs: the neurodisability scale. J Vet Intern Med. 2023;37:1111–1118. 10.1111/jvim.16717 PMC10229334 37092590

